# Exosomes derived from miR-338-3p-modified adipose stem cells inhibited inflammation injury of chondrocytes via targeting RUNX2 in osteoarthritis

**DOI:** 10.1186/s13018-022-03437-2

**Published:** 2022-12-26

**Authors:** ChunLiang Li, Wei Li, GengZang Pu, JingWen Wu, Feng Qin

**Affiliations:** 1 Department of Orthopedic, Qinghai Provincial People’s Hospital, Xining, 810006 Qinghai China; 2 Department of Emergency Surgery, Qinghai Provincial People’s Hospital, Xining, 810006 Qinghai China; 3 Department of Endocrinology, Qinghai University Affiliated Hospital, Chengxi District, No. 6, Xichuan South Road, Xining, 810006 Qinghai China

**Keywords:** Osteoarthritis, Exosomes, miR-338-3p, Mesenchymal stem cells from adipose tissue

## Abstract

**Background:**

Osteoarthritis (OA) is a chronic degenerative disease that is one of the main causes of disability in middle-aged and elderly people. Adipose stem cell (ASC)-derived exosomes (ASC-Exo) could repair cartilage damage and treat OA. MiRNA-338-3p expression was confirmed to play a role in inhibiting proinflammatory cytokines. Herein, we aimed to explore the mechanism by which exosomes derived from miR-338-3p overexpressing ASCs protects chondrocytes from interleukin (IL)-1β-induced chondrocyte change.

**Methods:**

Exosomes were extracted from ASCs transfected with miR-338-3p or its antisense inhibitor. The ASC-Exos (miR-338-3p silencing/overexpression) were incubated with IL-1β-induced ATDC5 cells, followed by evaluation of the chondrocyte proliferation, degradation, and inflammation injury.

**Results:**

In vitro results revealed that ASC-Exos inhibited the expression of prostaglandin E2 (PGE2), IL-6, IL-1β, and TNF-α, as well as promoted the proliferation of ATDC5 cells. Moreover, ASC-Exos inhibited inflammation injury and degradation of ATDC5 cells by transferring miR-338-3p. Luciferase reporter assays showed that RUNX2 was a target gene of miR-338-3p. Additionally, RUNX2 overexpression in ATDC5 cells reversed the protective effect of miR-338-3p on chondrocytes. Taken together, this study demonstrated that exosomes secreted from miR-338-3p-modified ASCs were effective in the repair of IL-1β-induced chondrocyte change by inhibiting RUNX2 expression.

**Conclusions:**

Our result provided valuable data for understanding the mechanism of ASC-Exos in OA treatment.

**Graphical abstract:**

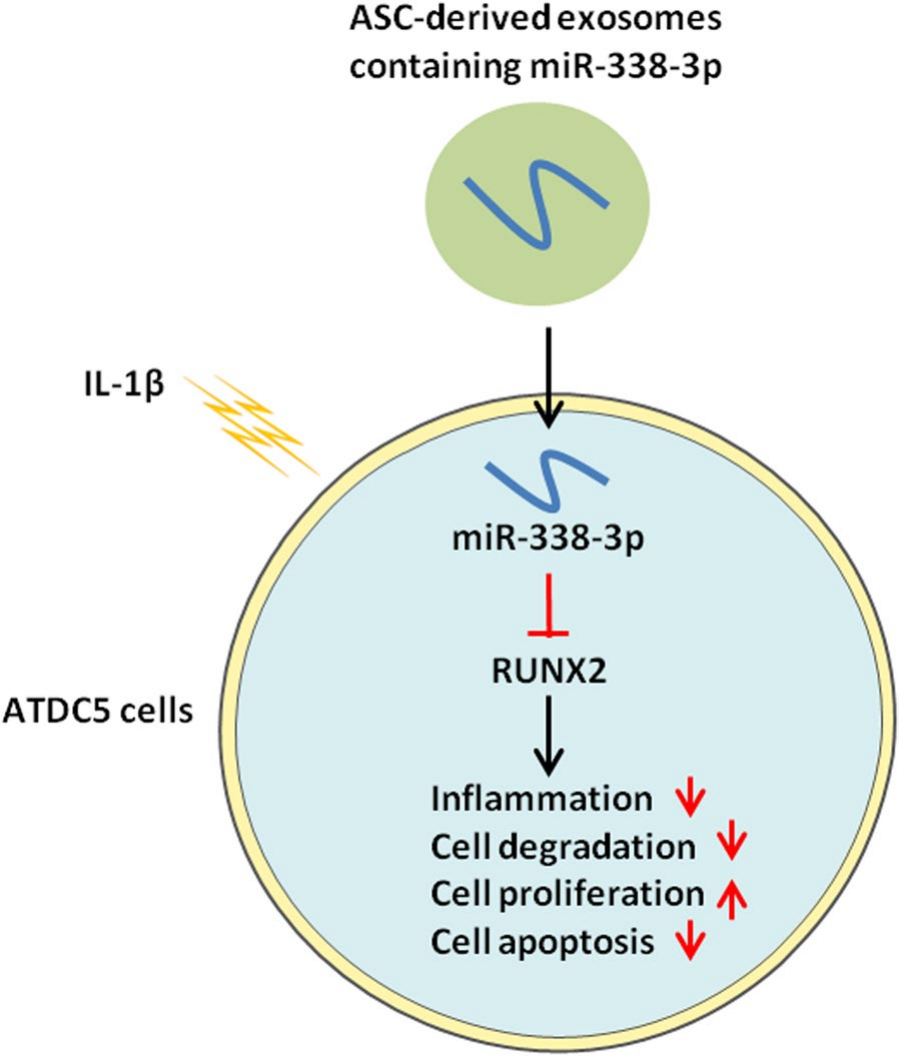

## Background

Osteoarthritis (OA) is a posttraumatic or age-related degenerative disease that is mainly characterized by the progressive degradation of cartilage with tissue inflammation, along with joint pain and stiffness [1–3]. Recent studies showed that inflammatory mediators played important roles in the process of OA progression [4, 5]. Up-regulated interleukin (IL)-6, IL-1β, tumor necrosis factor-alpha (TNF-α), and NO have been found in multiple niches in the joint, including subchondral bone, cartilage, synovial fluid, and synovial membrane of OA patients [6]. In particular, IL-1β could strongly inhibit chondrocyte differentiation and proliferation, as well as promote cell apoptosis and cartilage degeneration in OA progression [7]. IL-1β also promoted the expression of proteases involved in the destruction of cartilage matrix, including matrix metalloproteinases (MMPs) and aggrecanase, thereby accelerating the degradation of type II collagen and proteoglycans [8].

Mesenchymal stem cells (MSCs) are a type of pluripotent stem cells derived from the early mesoderm and ectoderm [9]. MSCs exist in the bone marrow, fat, synovial, umbilical cord and muscle, and so on [9]. Recent studies confirmed that MSCs regulated the cellular microenvironment through cell–cell interaction and the secretion of various cytokines, and then repaired tissue damage and other pathological processes [10, 11]. Especially, MSCs were suggested to be the primary drivers of cartilage repair [12]. Clinical research showed that intra-articular injections of human adipose-derived mesenchymal stem cells (haMSCs) effectively improved pain, function, and cartilage volume of the knee joint [12]. Adipose stem cells (ASCs) are capable of directed differentiation to adipocytes, chondrocytes, osteoblasts, endothelial cells, cardiac myocytes, and so on [13]. Previous research showed in a mouse model of OA, the injection of ASCs into the knee suppressed synovial thickening and repaired cartilage damage [14]. Due to the properties-ease of the harvest of adipose tissue, high homology and low donor site morbidity-ASCs have been widely used in research on the treatment of diseases including OA [15].

Exosomes are nanoscale vesicles secreted by cells, with a diameter range from 40 to 100 nm [16]. Exosomes bear various biological molecules including proteins, lipids, RNAs, and transport these cargos between different cell types, thus mediating intercellular communication [16, 17]. As an important means of paracrine, exosomes can be involved in the process of cell proliferation, migration, differentiation, and so on [18]. Previous studies showed that MSC-derived exosomes (MSC-Exos) participated in a variety of pathophysiological processes and were effective in repairing tissue damage [19, 20]. Emerging research indicated that MSC-exos could regulate cartilage regeneration by suppressing chondrocyte hypertrophy and stimulating angiogenesis [21]. However, the regulation mechanism of ASC-derived exosomes (ASC-Exos) on cartilage regeneration and bone metabolism has not been reported yet.

MicroRNAs (miRNAs) are 21–25 nucleotide non-coding RNA molecules that post-transcriptionally regulate gene expression through binding to the 3’-untranslated regions (3’-UTRs) of mRNA, which are involved in multiple cell activities [19, 22, 23]. According to extensive research, miRNAs in the exosomes play an important role [16, 24, 25]. It was worth noticing that miR-338-3p was found in ASC-Exos previously [26]. Nevertheless, the potential role of exosomal miR-338-3p in OA has not yet been elucidated. Therefore, we aimed to explore the therapeutic potential of miR-338-3p derived from ASC-Exo and its mechanisms of action in OA.

## Methods

### Animals

C57BL/6 male mice (SPF grade, 10–12 weeks) were purchased from Dashuo Animal Experiment Co., Ltd. (Chengdu, Sichuan) and raised in the Qinghai Provincial People's Hospital. The feeding environment was 25 ± 1 °C, relative humidity 50%-60%, and light/darkness for 12 h circulation. The mice are allowed to eat and drink freely. The study protocols were approved by the Ethics Committee of Qinghai Provincial People's Hospital (number: QHPH-20190823).

### Cell culture

Mouse ASCs were isolated and cultured as previously described [27]. Briefly, the C57BL/6 male mice were anesthetized with 1% sodium pentobarbital (50 mg/kg) and euthanized. The adipose tissue was obtained from the mouse groin and the surface fascia and blood vessels were removed. Small pieces were incubated with 0.2 mg/ml type I collagenase for 3 h at 37 °C in 5% CO_2_ then neutralized with complete DMEM containing 10% fetal bovine serum (FBS, Sigma-Aldrich, St. Louis, MO, USA) and 5 ng/ml basic fibroblast growth factor (PeproTech, Rocky Hill, NJ). The cell pellet was collected by centrifuging at 2749*g* for 10 min and resuspended with complete DMEM containing 10% FBS. The cells were seeded in a 25 cm^2^ culture flask and cultured in a 37 °C, 5% CO_2_ incubator after being filtered with a 200-mesh sieve. After 24 h, the adherent fraction was resuspended in a fresh medium. The medium was changed every 3 days thereafter until the cells grow to 80–90% confluency. ASCs in the three passages were used for the experiments. For some experiments, ASCs were treated with an inhibitor of exosome biogenesis/release (GW4869, 10 μM) for 48 h.

The murine chondroprogenitor cell line ATDC5 was purchased from the Type Culture Collection of the Chinese Academy of Sciences (Shanghai, China). Cells were maintained in the RPMI-1640 medium (Hyclone, Logan, UT, USA), 10% FBS, and 1% penicillin/streptomycin (v/v). Cells were grown in a humidified atmosphere of 5% CO_2_ at 37 °C. The RPMI-1640 medium was replaced every 2 days. For some experiments, non-contact co-culture of ATDC5 cells and ASCs were performed in cell culture inserts (24 wells, 0.4 μm pore size, Falcon, Franklin Lakes, NJ, USA). ASCs (1 × 10^4^ cells/well) and ATDC5 cells (5 × 10^4^ cells/well) were seeded into upper and lower chambers, respectively. The ATDC5 cells were pretreated with ASC-Exo for 2 h and then stimulation with or without proinflammatory factor IL-1β (10 ng/mL). For protein expression analysis, the samples were harvested at 48 h.

### Cell transfection

Lipofectamine 3000 transfection reagent was used for the transient transfection of NC-mimic/miR-338-3p mimic/NC inhibitor/miR-338-3p inhibitor into ASCs (1.0 × 10^5^ cells/well) and transient transfection of pcDNA-RUNX2 into ATDC5 cells (1.0 × 10^5^ cells/well) according to the manufacturer's instructions.

### Identification of ASCs

ASCs (passage 3) were verified using flow cytometry to assess the phenotypic characteristics. ASCs were grown to 80–90% confluency, trypsinized, and resuspended in PBS (Invitrogen, Carlsbad, CA, USA) and adjusted the cell concentration to 1.0 × 10^6^ cells/ml. ASCs were identified using flow cytometry and induced differentiation as described previous references [28, 29]. ASCs were stained with corresponding antibodies of CD29, CD90, CD105, CD34, and CD45. Cells were then analyzed by a Beckman-Coulter Epics XL (Beckman-Coulter, Miami, FL). Flow Jo software (Tree Star, OR, USA) was used to analyze the results. The multi-differentiation potential of ASCs was evaluated through Oil red O (adipogenic differentiation), Alizarin red (osteogenic differentiation), alkaline phosphatase (osteogenic differentiation), and Alcian blue stains (chondrogenic differentiation), respectively, and observed in an EVOS XL Core microscope (bright-field; Invitrogen, Carlsbad, CA, USA).

### Isolation of ASC-derived exosomes

ASC-Exos were extracted by differential ultracentrifugation. Briefly, ASCs (passage 3) were cultured with complete DMEM containing 10% FBS (depletion of exosomes by ultracentrifugation) for 2 days. The cell supernatants was centrifuge at 300 × g for 10 min at 4 °C, 1200×*g* for 20 min at 4 °C, and 10,000×*g* for 30 min at 4 °C to eliminate dead cells and large cell debris. II The supernatants were then collected and ultracentrifuged (Hitachi, CP100NX, Japan) at 100,000×*g* for 70 min at 4 °C, followed by being washed in PBS, and ultracentrifuged at 100,000×*g* for 70 min at 4 °C to eliminate contaminating proteins. Final pellets were resuspended in sterile 100 μl PBS and characterized by nanosight tracking analysis system (Brookhaven Instruments Corp, USA), transmission electron microscopy (JEM-1400FLASH; JEOL Ltd., Tokyo, Japan), and Western blot.

### Transmission electron microscopy (TEM)

The morphology of ASC-Exos in purified samples was observed using TEM. The enriched exosomes (15 μl) were placed onto a carbon-coated copper TEM grid and incubated for 10 min. With the excess fluid removed, the samples were then stained with 2% phosphotungstic acid (pH = 6.5) for 1–2 min at room temperature and then subjected to TEM.

### CCK-8 assay

The cell vitality of ASCs and ATDC5 cells was measured using the Cell Counting Kit 8 (CCK-8, Thermo Fisher Scientific) according to the manufacturer’s instructions. Absorbance was recorded at 450 nm.

### Flow cytometry assay

The apoptosis of ATDC5 cells was analyzed using an Annexin V- according to the manufacturer’s protocol. Briefly, the cells were washed with PBS (Invitrogen, Carlsbad, CA, USA), and adjusted the cell concentration to 1.0 × 10^6^ cells/mL. The cells were subsequently suspended in a 150 μl buffering solution. Subsequently, the cells were stained with 10 μg/ml Annexin V-FITC and 5 μl PI at 4 °C for 20 min in darkness. Apoptotic cells were then analyzed by a BD FACSCelesta™ Flow Cytometer (Becton, Dickinson, and Company).

### Immunofluorescence (IF)

The expression of aggrecan and Col2a1 in ATDC5 cells was estimated by IF. Briefly, ATDC5 cells were fixed with 4% paraformaldehyde for 15 min and permeabilized with 0.01% Triton X-100 for 10 min, and then blocked with 5% normal goat serum for 1 h at room temperature. The cells were incubated with an anti-aggrecan (Servicebio, Wuhan, China; GB11373; 1/500) and anti-Col2a1(Abcam, Cambridge, MA, USA; ab34712; 1/100) overnight at 4 °C, washed with PBS, and then incubated with FITC-conjugated secondary antibody in the dark for 2 h at room temperature. The nuclei of the cells were stained with DAPI. All samples were analyzed using fluorescence microscopy (Nikon TE2000 microscope, Nikon, Tokyo, Japan). Fluorescence intensity was performed with ImageJ software.

#### Quantitative RT‐qPCR

The total RNAs of ATDC5 cells were isolated employing TRIzol® reagent (Thermo Fisher, Massachusetts, USA). cDNA was gained by reverse transcription kit (Invitrogen, Carlsbad, CA, US). The relative levels of target gene RNA transcriptome were obtained by RT-qPCR by using an SYBR Premix Ex Taq kit (Bao Biological Engineering, Dalian, China). The reverse transcriptional reaction conditions were as follows: 95 °C for 30 s, 40 cycles of 95 °C for 5 s, and 60 °C for 30 s. The relative gene expression level was determined by applying the 2^−△△Ct^ method on ABI software, Foster City, CA. RT-qPCR primer sequences are as follows: miR-338-3p forward- GGG TCC AGC ATC AGT GAT T, miR-338-3p reverse-GCG TTG TGT TGT GTT GTG TT; RUNX2 forward-GGA GTG GAC GAG GCA AGA GT, RUNX2 reverse-AAT CAC TGA GGC GGT CAG AG; β-actin forward-CTC CAT CCA CCG CAA ATG CTT CT, β-actin reverse-GCTCCA ACC GAC TGC TGT CAC CTT C; U6 forward-CTC GCT TCG GCA GCA CA, U6 reverse-AAC GCT TCA CGA ATT TGC GT.

### Western blot analysis

ASC-Exos or ATDC5 cells were resuspended in RIPA buffer (Signaling Technology, Inc.). The concentration of protein was determined by a BCA kit (Sigma-Aldrich; Merck KGaA). Total protein (30 µg/lysates) was separated via 10% SDS-PAGE. And then the separated proteins were transferred to nitrocellulose membranes. The membranes were blocked with 5% nonfat dried milk overnight at 4 °C and incubated with the following corresponding protein antibodies: CD9 (Abcam, Cambridge, MA, USA; ab236630; 1/1000), CD63 (Abcam, Cambridge, MA, USA; ab231975; 1/1000), MMP3 (Abcam, Cambridge, MA, USA ab52915; 1/1000), MMP13 (Abcam, Cambridge, MA, USA; ab390112; 1/3000), aggrecan (Abcam, Cambridge, MA, USA; ab3778; 1/1000), Col2a1(Abcam, Cambridge, MA, USA; ab34712; 1/1000), Runx2 (Abcam, Cambridge, MA, USA; ab76956; 1/3000). Then, the membranes were washed with Tris-buffered saline/0.1% Tween (TBST) and incubated for 1.5 h with an HRP Goat anti-Rabbit IgG (Abcam, ab6721). The bands were visualized using the ECL system (Affinity Biosciences, Cincinnati, Ohio, USA). The net optical density was measured using Quantity One software (Bio-Rad).

### Enzyme-linked immunosorbent assay (ELISA)

The contents of prostaglandin E2 (PGE2), IL-6, IL-1β, and TNF-α in culture supernatants of ATDC5 cells were measured with ELISA kits (Takara, Japan), following the manufacturer’s instructions. The absorbance was measured at 450 nm wavelength and was estimated using an enzyme-linked immune monitor (Thermo Fisher Scientific, Inc., USA).

### Luciferase activity assay

The fragments of the 3′-untranslated region (UTR) of RUNX2 containing the predicted wild-type (WT) binding sites of miR-338-3p or mutated miR-338-3p binding sites (MUT) were amplified by PCR and inserted into a pMIR-REPORT luciferase reporter vector (Ambion, Austin, TX, USA), named as RUNX2-3’UTR-WT and RUNX2-3’UTR-MUT. Afterward the luciferase reporter gene plasmid and miR-338-3p mimic or NC mimic were co-transfected into HEK-293T cells (4 × 10^4^ cells/well) using the Lipofectamine 3000 (Invitrogen, Carlsbad, CA, USA). After transfection for 24 h, the relative luciferase activity was detected by a luciferase reporter assay system.

### Statistical analysis

The data were represented as means ± standard deviation. Statistical analysis was performed using SPSS 20.0 (IBM Corp.). One-way analysis of variance (ANOVA) with Tukey’s post hoc test of means was used for comparison between groups. Differences with a *P* < 0.05 were considered to indicate statistically significant.

## Results

### Isolation and identification of ASCs

**Fig. 1:**
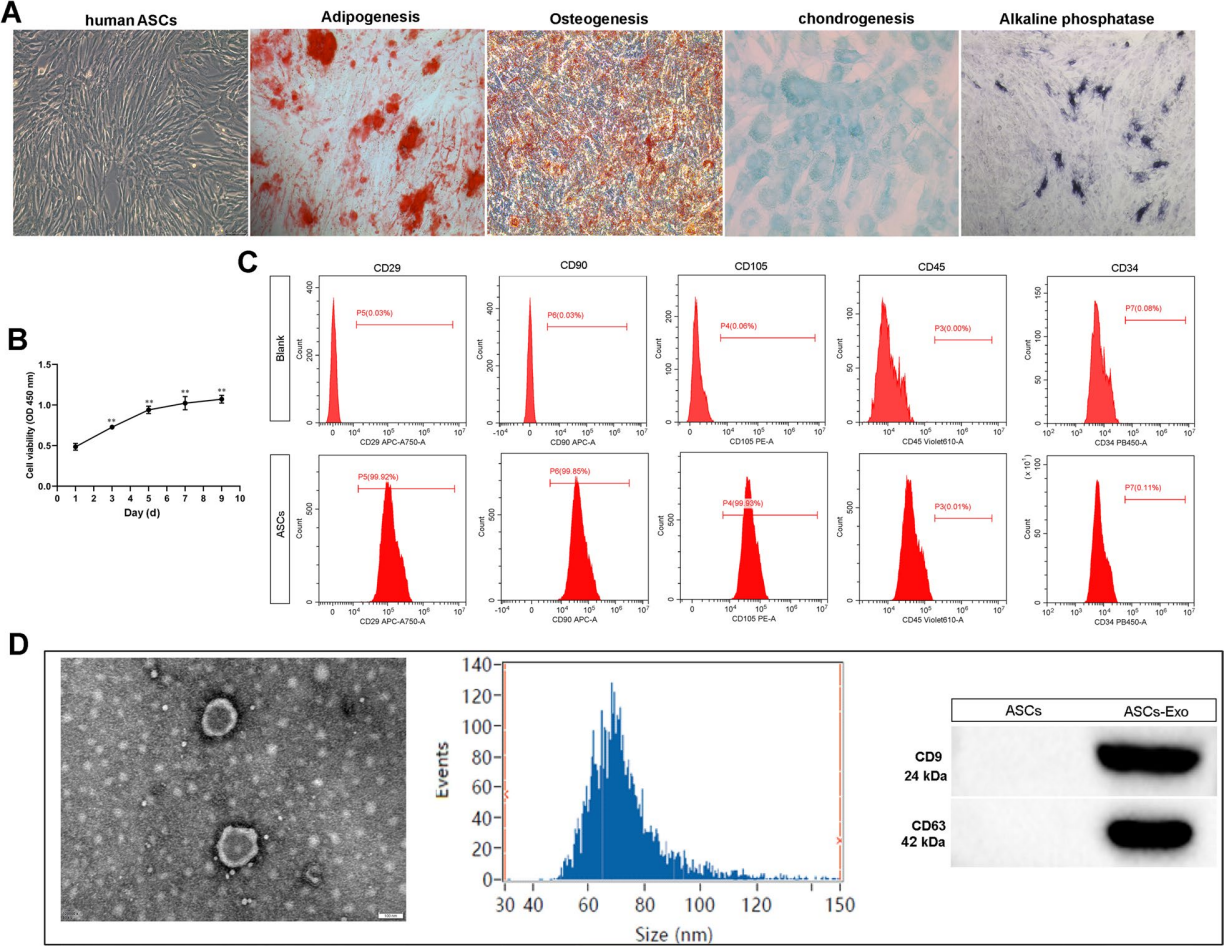
Classification of adipose stem cells (ASCs) and exosomes. **A** ASCs exhibited a representative spindle-like morphology (scale bar: 100 μm). ASCs were stained with Oil red O, Alizarin red, alkaline phosphatase, and Alcian blue stain. **B** The cell proliferation of ASCs was tested by CCK-8 assay. ^**^P < 0.01 (VS. 1 day). **C** Flow cytometric analysis of characteristic cell surface markers of ASCs (CD29, CD90, CD105, CD45, and CD34). **D** Morphology of exosomes observed by transmission electron microscopy (TEM). Scale bar: 100 nm. Particle size distribution of exosomes was measured by Nanosight. The expression of exosome surface markers (CD9 and CD63) was measured using western blot

ASCs were isolated from adipose tissue in the mouse groin as described in the Methods section. In passage 3 (P3), ASCs were identified and used in subsequent experiments. Oil red O stain showed adipogenesis in ASCs after adipogenic induction (Fig. 1A). The presence of red calcium nodules after the Alizarin Red stain demonstrated osteogenic differentiation (Fig. 1A). Alcian Blue stain demonstrated chondrogenic differentiation (Fig. 1A). And CCK-8 assay showed that the cell viability of ASCs was significantly increased during 10 days of continuous culture (Fig. 1B). Meanwhile, ASCs were characterized by flow cytometry after staining surface markers (Fig. 1C). These cells were found to be positive for CD29, CD90, and CD105, but did not express the CD45 and CD34 (Fig. 1C).

### ASC-Exo identification

TEM showed that these vesicles were hollow, spherical microvesicles (Fig. 1D). The data from Nanosight assay indicated that the diameter range of these vesicles was 50–110 nm and the main peak of particle size was located at 68.3 nm, which was in agreement with the characteristics of exosomes (Fig. 1D) [30]. Then, the expression of surface exosomal markers CD9 and CD63 was verified by western blot analysis. The results showed that ASC-Exo carried large amounts of CD9 and CD63 compared with ASC lysates (Fig. 1D). Overall, we successfully obtained exosome particles from ASCs.

### ASC-Exo inhibited IL-1β-induced inflammation and degradation of chondrocytes

**Fig. 2:**
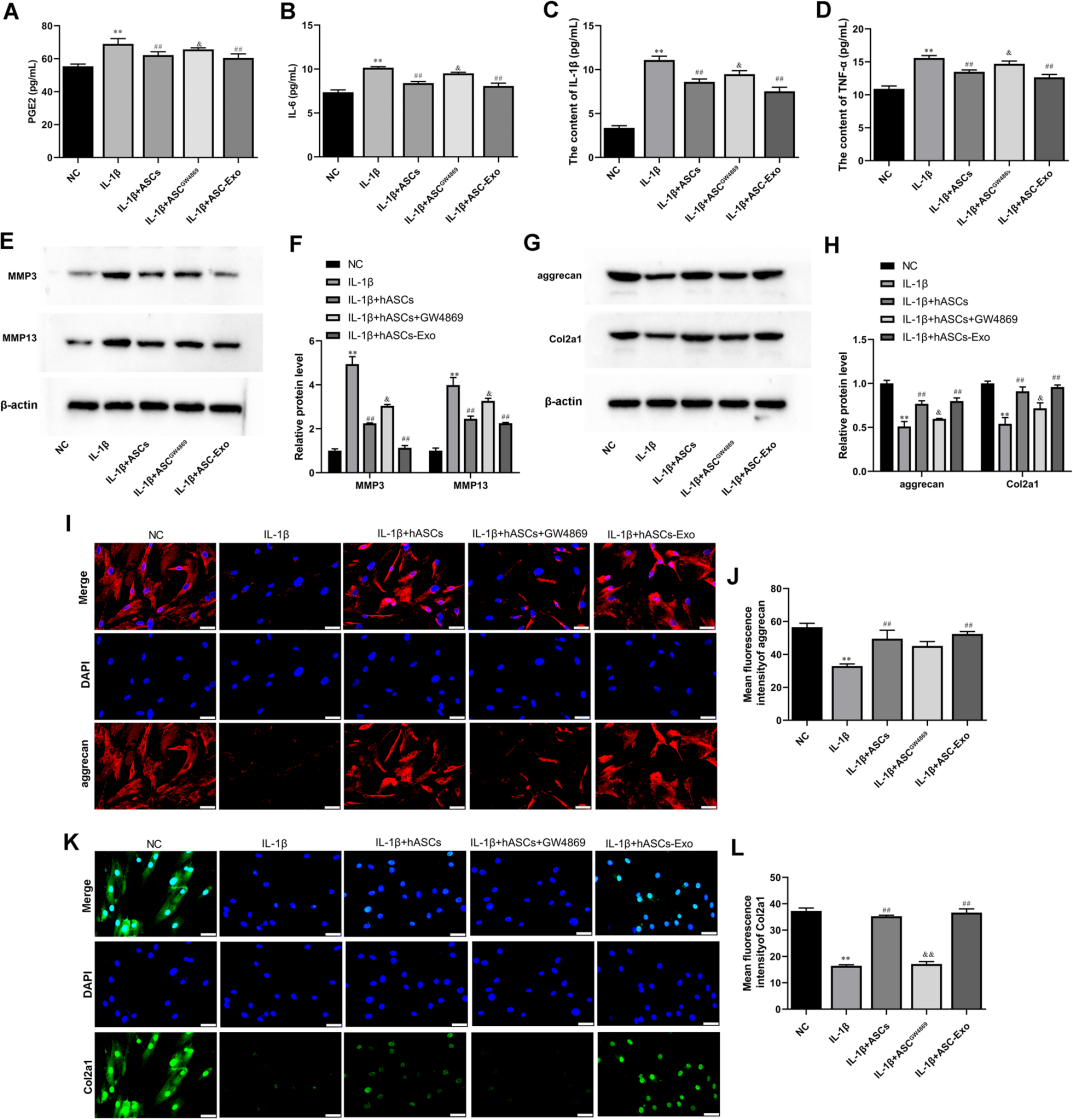
ASC-Exo promoted IL-1β-reduced cell proliferation and inhibited IL-1β-induced cell apoptosis of ATDC5 cells and in delivering miR-338-3p. **A–D** The expression levels of PGE2, IL-6, IL-1β, and TNF-α in ATDC5 cell supernatant were detected by ELISA. **E** and **F** Gene expression level of MMP3 and MMP13 in ATDC5 cells was determined by Western blot analysis. β-actin was used as the internal reference gene. **G** and **H** The expression of aggrecan and Col2a1 was assayed by Western blot analysis. β-actin was used as the internal reference gene. **I–L** Immunofluorescence (IF) was used to test aggrecan and Col2a1 expression in ATDC5 cells. Nuclei were visualized using DAPI. Scale bar: 50 μm.^**^P < 0.01 (VS. NC), ^##^*P* < 0.01 (VS. IL-1β), ^&^*P* < 0.05 (VS. IL-1β + ASCs), ^&&^*P* < 0.01 (VS. IL-1β + ASCs). ASC^GW4869^, ASCs treated with GW4869; ASC-NC^−^, ASCs treated with NC inhibitor; ASC-338^−^, ASCs treated with miR-338-3p inhibitor; ASC-NC^+^, ASCs treated with NC mimic; ASC-338^+^, ASCs treated with miR-338-3p mimic

WE determined whether ASC-Exo could affect the IL-1β-induced expression of inflammatory factors IL-6, IL-1β, and TNF-α in chondrocytes. ATDC5 cells were pretreated with ASC-Exo for 2 h and then stimulated with IL-1β for 24 h. Moreover, other ATDC5 cells were co-cultured with ASCs which were treated with or without GW4869. ELISA data found that IL-1β treatment significantly increased the expression of PGE2, IL-6, IL-1β, and TNF-α as compared with untreated control ATDC5 cells (Fig. 2A–D). Levels of these inflammatory factors were decreased in ATDC5 cells after co-culture with ASCs or ASCs-Exos (Fig. 2A–D). However, supplementation of the exosome secretion blocker GW4869 significantly hindered the weakening effect of ASCs on inflammation (Fig. 2A–D).

It was known that MMP3 and MMP13 played a key role in the inhibition of proteoglycan synthesis and stimulation of matrix degradation in chondrocytes [4]. Western blot analysis also proved the induction of MMP3 and MMP13 expression in IL-1β-treated ATDC5 cells (Fig. 2E and F). Interestingly, pretreatment of ATDC5 cells with ASCs and ASC-Exos both significantly inhibited the expression of MMP3 and MMP13 (Fig. 2E and F). Meanwhile, GW4869 treatment reversed the decrease of MMP3 and MMP13 in ASCs and ASC-Exos-treated ATDC5 cells (Fig. 2E and F). Col2a1 (type II collagen alpha 1) and aggrecan are major cartilage extracellular matrix proteins that are important for normal cartilage function [31]. Western blot and IF demonstrated that IL-1β induction decreased the expression of Col2a1 and aggrecan, which were promoted by ASCs and ASC-Exos (Fig. 2G and H). Moreover, the GW4869 treatment reversed the increases of Col2a1 and aggrecan induced by ASC_S_ (Fig. 2G and H)_._

### ASC-Exo promoted IL-1β-reduced cell proliferation and inhibited IL-1β-induced cell apoptosis of ATDC5 cells and in deliver miR-338-3p

**Fig. 3:**
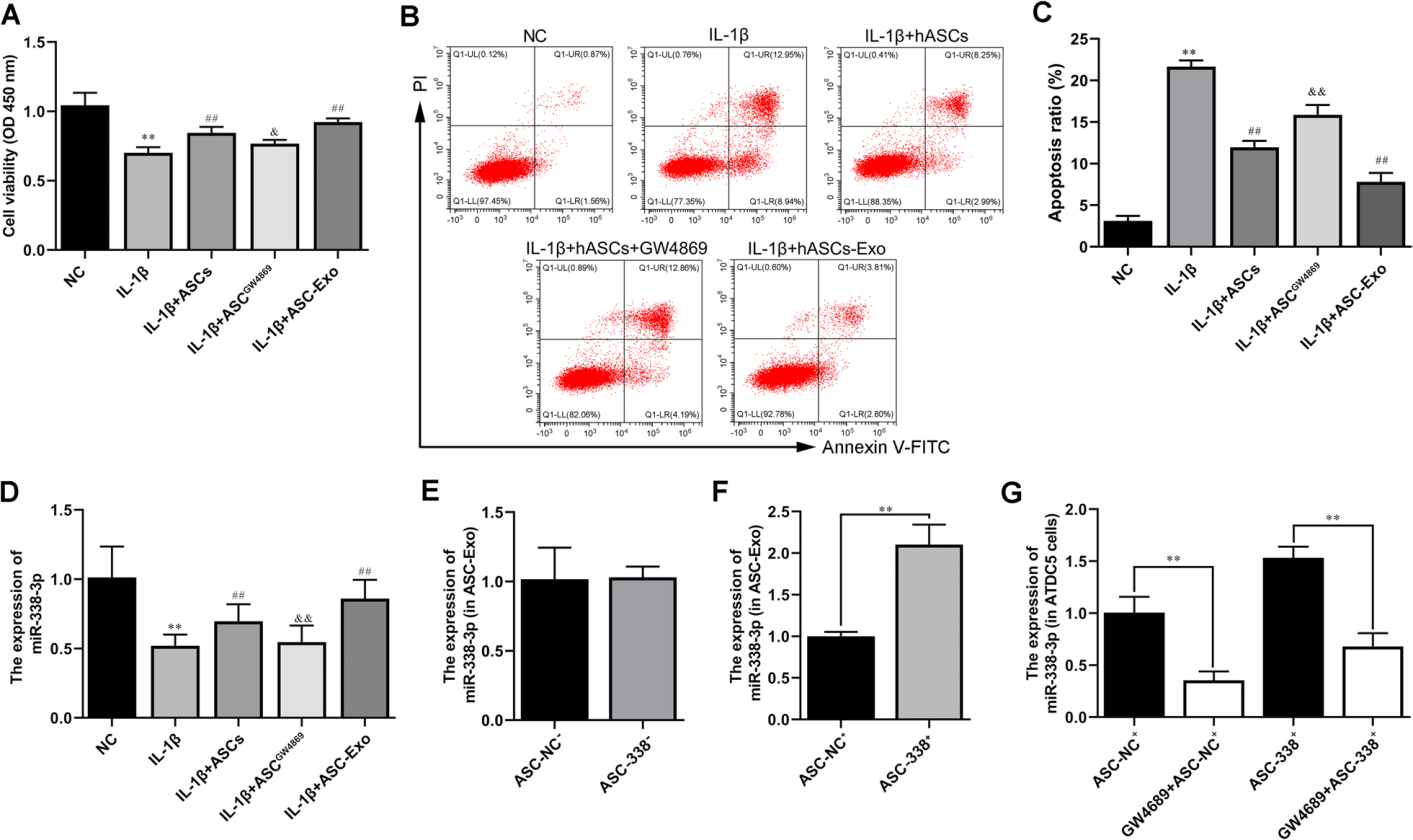
ASC-Exo promoted IL-1β-reduced cell proliferation and inhibited IL-1β-induced cell apoptosis of ATDC5 cells and in delivering miR-338-3p. **A** The cell proliferation of ATDC5 cells was tested by CCK-8 assay. **B** and **C** Flow cytometry analysis of apoptosis in ATDC5 cells. **D** The expression level of miR-338-3p in ASC-Exos **(E** and **F)** and ATDC5 cells **(G)** was determined by RT-qPCR. ^**^P < 0.01 (VS. NC), ^##^*P* < 0.01 (VS. IL-1β), ^&^*P* < 0.05 (VS. IL-1β + ASCs), ^&&^*P* < 0.01 (VS. IL-1β + ASCs). ASC^GW4869^, ASCs treated with GW4869; ASC-NC^−^, ASCs treated with NC inhibitor; ASC-338^−^, ASCs treated with miR-338-3p inhibitor; ASC-NC^+^, ASCs treated with NC mimic; ASC-338^+^, ASCs treated with miR-338-3p mimic

In addition, the CCK-8 results showed that ASC_S_ and ASC-Exos inhibited the decreased-cell proliferation of ATDC5 cells caused by IL-1β (Fig. 3A). And GW4869 treatment blocked the increase of cell proliferation induced by ASC_S_ (Fig. 3A). Meanwhile, flow cytometry analysis also proved that IL-1β induction increased the cell apoptosis of ATDC5 cells, which was inhibited by ASCs and ASC-Exos (Fig. 3B and C). GW4869 treatment blocked reversed the decrease of cell apoptosis induced by ASC_S_ (Fig. 3B and C). Importantly, the level of miR-338-3p was reduced by IL-1β, which was markedly induced by ASCs and ASC-Exos treatment (Fig. 3D). To further investigate whether ASC-Exos mediated miR-338-3p transfer, the expression of miR-338-3p in ATDC5 cells was measured after ASCs were treated with GW4869. As shown in Fig. 3D, the levels of miR-338-3p in ATDC5 cells co-cultured with GW4869 treated-ASCs (ASC^GW4869^) were significantly decreased compared with ATDC5 cells co-cultured with untreated ASCs (Fig. 3D). In addition, the miR-338-3p inhibitor was used to treat ASCs to suppress the function of miR-338-3p. RT-qPCR showed no change in the expression level of miR-338-3p in exosomes of miR-338-3p inhibitor-treated-ASCs (ASC-338^−^). Meanwhile, the expression level of miR-338-3p was increased in the exosomes of miR-338-3p mimic transfected-ASCs (ASC-338^+^) (Fig. 3D). Next, ATDC5 cells were co-cultured with ASC-338^+^ or GW4869-treated-ASC-338^+^. And the NC mimic treatment was used as a control group. The data showed that GW4869 treatment decreased the expression of miR-338-3p in ATDC5 cells, indicating that exosomes mediated miR-338-3p transportation between ASCs and ATDC5 cells (Fig. 3G).

### MiR-338-3p derived from ASC-Exos reduced inflammation and degradation of chondrocytes

**Fig. 4:**
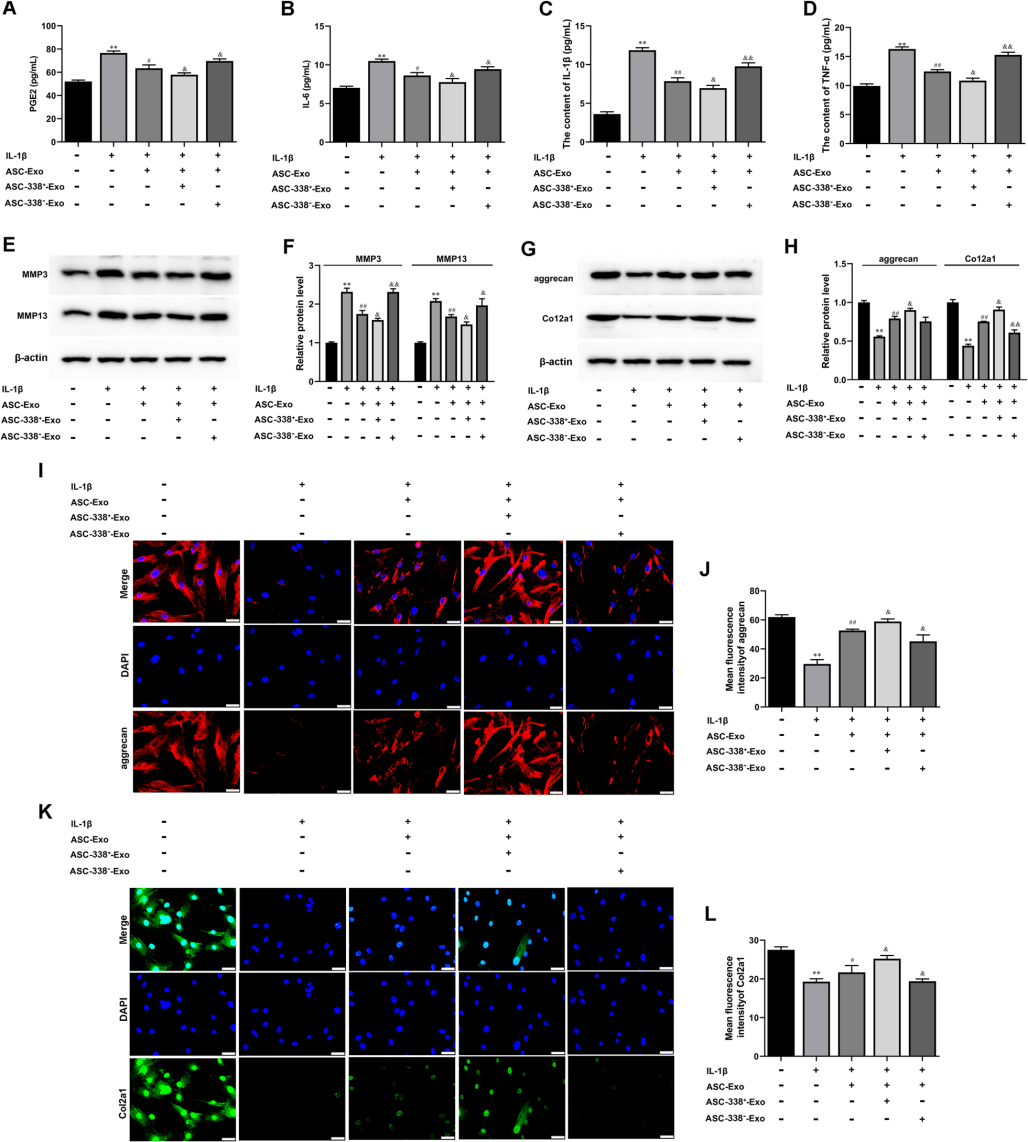
MiR-338-3p derived from ASC-Exos reduced inflammation and degradation of chondrocytes. **A–C** The expression levels of PGE2, IL-6, IL-1β, and TNF-αin ATDC5 cell supernatant were detected by ELISA. **D** Gene expression level of MMP3, MMP13, aggrecan, and Col2a1 in ATDC5 cells was determined by Western blot analysis. β-actin was used as the internal reference gene. **I–L** Immunofluorescence (IF) was used to test aggrecan and Col2a1 expression in ATDC5 cells. Nuclei were visualized using DAPI. Scale bar: 50 μm. ^*^P < 0.05 (VS. Blank), ^**^P < 0.01 (VS. Blank), ^#^*P* < 0.05 (VS. IL-1β), ^&^*P* < 0.05 (VS. IL-1β + ASC-Exo). ASC-338^+^-Exo, exosomes derived from miR-338-3p mimic-treated ASCs, ASC-338^−_^Exo, exosomes derived from miR-338-3p inhibitor-treated ASCs

The ASC-338^+^-Exo and ASC-338^−^-Exo were then used for subsequent experiments. The experimental results showed that ASC-Exos suppressed PGE2, IL-6, IL-1β, and TNF-α expression and that this effect could be blocked by miR-338-3p inhibitor and be strengthened by miR-338-3p mimic (Fig. 4A–D). Meanwhile, ASC-Exo decreased MMP3 and MMP13 expression in IL-1β-induced ATDC5 cells, which was further inhibited by ASC-338^+^-Exo and was increased by ASC-338^−^-Exo (Fig. 4E and F). Western blot and IF suggested that Col2a1 and aggrecan expression was enhanced by ASC-Exo in IL-1β-induced ATDC5 cells. ASC-338^+^-Exo further strengthened and ASC-338^−^-Exo weakened Col2a1 and aggrecan in IL-1β and ASC-Exo co-treated ATDC5 cells (Fig. 4G–L).

### MiR-338-3p derived from ASC-Exos stimulated cell proliferation and inhibited cell apoptosis of IL-1β-inducedATDC5 cells

**Fig. 5:**
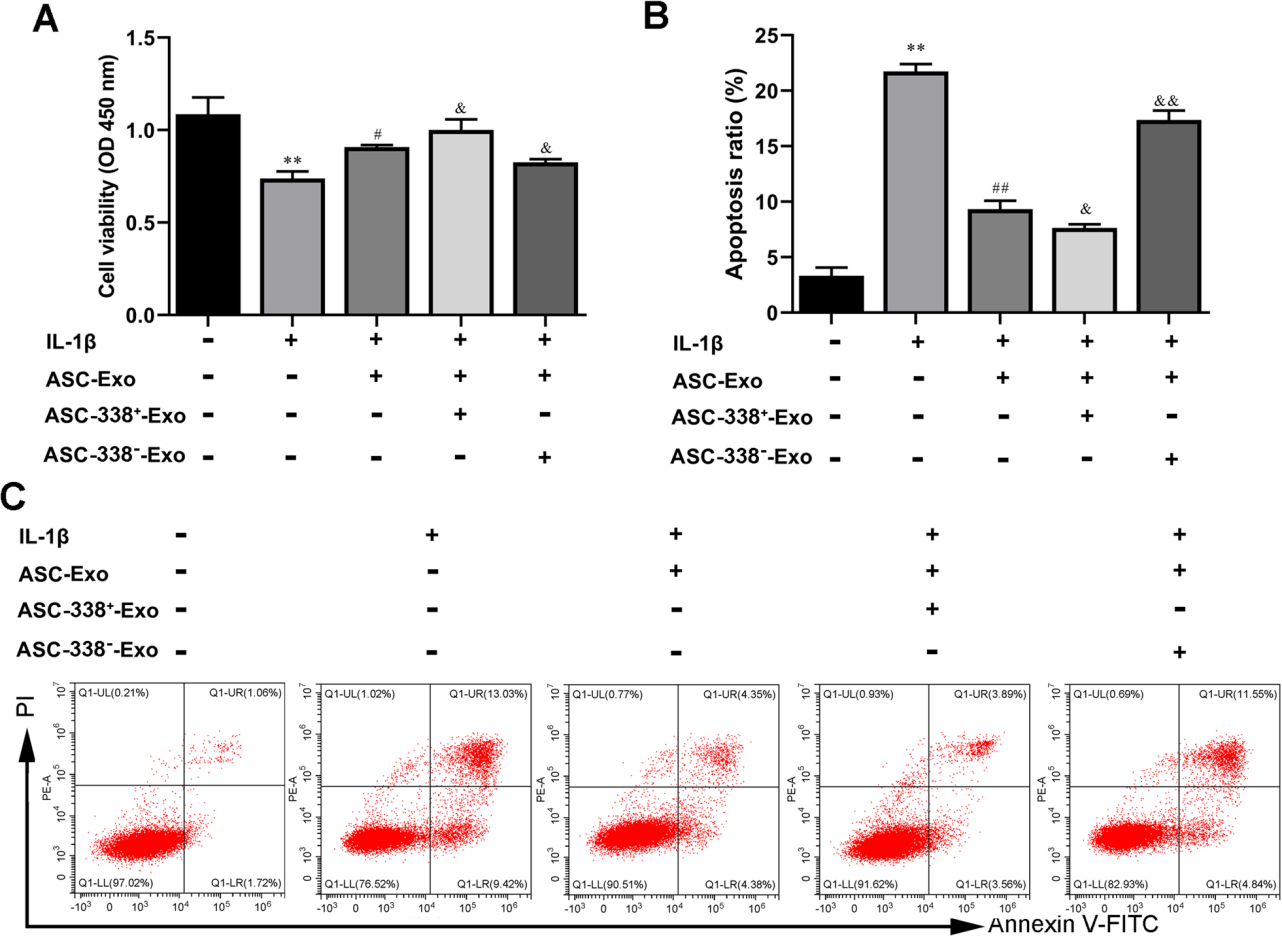
MiR-338-3p derived from ASC-Exos stimulated cell proliferation and inhibited cell apoptosis of IL-1β-inducedATDC5 cells. **A** The cell proliferation of ATDC5 cells was tested by CCK-8 assay. **B** and **C** Flow cytometry analysis of apoptosis in ATDC5 cells. ^*^P < 0.05 (VS. Blank), ^**^P < 0.01 (VS. Blank), ^#^*P* < 0.05 (VS. IL-1β), ^&^*P* < 0.05 (VS. IL-1β + ASC-Exo), ^&&^*P* < 0.01 (VS. IL-1β + ASC-Exo). ASC-338^+^-Exo, exosomes derived from miR-338-3p mimic-treated ASCs, ASC-338^−_^Exo, exosomes derived from miR-338-3p inhibitor-treated ASCs

In addition, the CCK-8 results showed that ASC-Exos promoted ATDC5 cell proliferation reduced by IL-1β, which was reversed by ASC-338^−^-Exo treatment (Fig. 5A). And ASC-338^+^-Exo promoted the proliferation more effectively than ASC-Exo treatment did (Fig. 5A). Flow cytometry analysis indicated that ASC-Exo inhibited cell apoptosis of IL-1β-induced ATDC5 cells. ASC-338^+^-Exo further reduced cell apoptosis of IL-1β and ASC-Exo co-treated ATDC5 cells (Fig. 5B and C). ASC-338^−^-Exo reversed cell apoptosis inhibited by ASC-Exos (Fig. 5B and C).

### MiR-338-3p derived from ASC-Exos targeted RUNX2 expression in ATDC5 cells

**Fig. 6:**
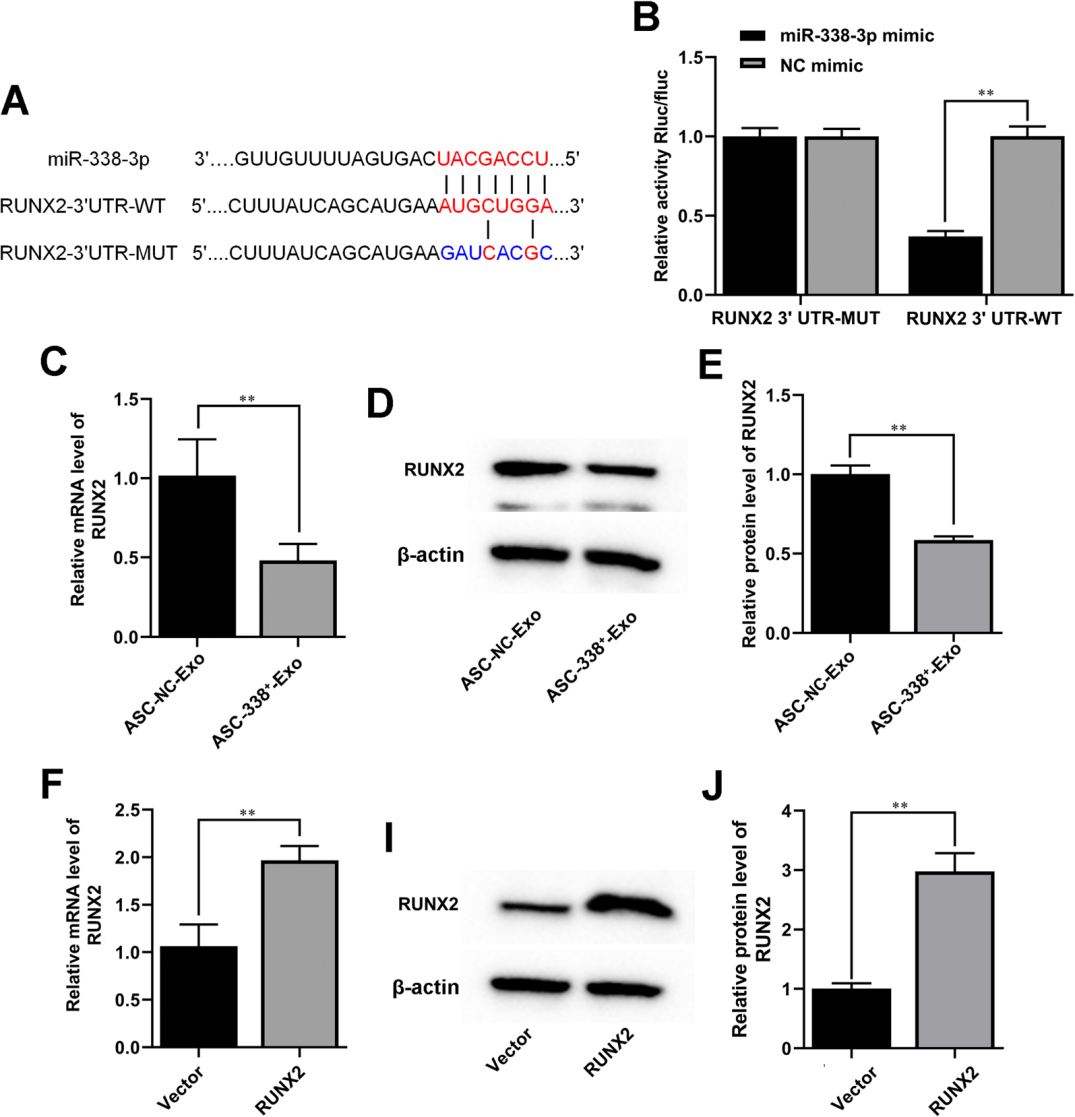
MiR-338-3p derived from ASC-Exos targeted RUNX2 expression in ATDC5 cells. **A** Alignment of miR-338-3p and the 3′-UTR of RUNX2, a potential miR-338-3p target. **B** A luciferase reporter carrying the 3’-UTR of wild-type (RUNX2 3′UTR-WT) or mutant (RUNX2 3′UTR-MUT) RUNX2 was introduced into HEK-293T cells along with NC mimic or miR-338-3p mimic. Gene expression level of RUNX2 in ATDC5 cells was determined by RT-qPCR (**C** and **D**) and Western blot analysis **(D–E, I–J)**. β-actin was used as the internal reference gene. ^**^P < 0.01 (VS. ASC-NC-Exo/vector). ASC-NC-Exo, exosomes derived from NC mimic-treated ASCs; ASC-338^+^-Exo, exosomes derived from miR-338-3p mimic-treated ASCs; RUNX2, pcDNA-RUNX2

The putative binding site of miR-338-3p to the 3′-UTR of RUNX2 was predicted using the TargetScan and Starbase v2.0 (Fig. 6A). The luciferase assay demonstrated that the luciferase activity of the wt pMIR-REPORT RUNX2 was attenuated by miR-338-3p (Fig. 6B). Our results further showed that ASC-338^+^-Exo significantly inhibited RUNX2 expression in ATDC5 cells (Fig. 6C–E). Next, for subsequent experiments, we established an efficient pcDNA-RUNX2 (RUNX2) to increase the expression of RUNX2 in ATDC5 cells (Fig. 6F–J).

### RUNX2 overexpression reversed ASC-338^+^-Exo-mediated attenuation of ATDC5 cell inflammation and degradation

**Fig. 7:**
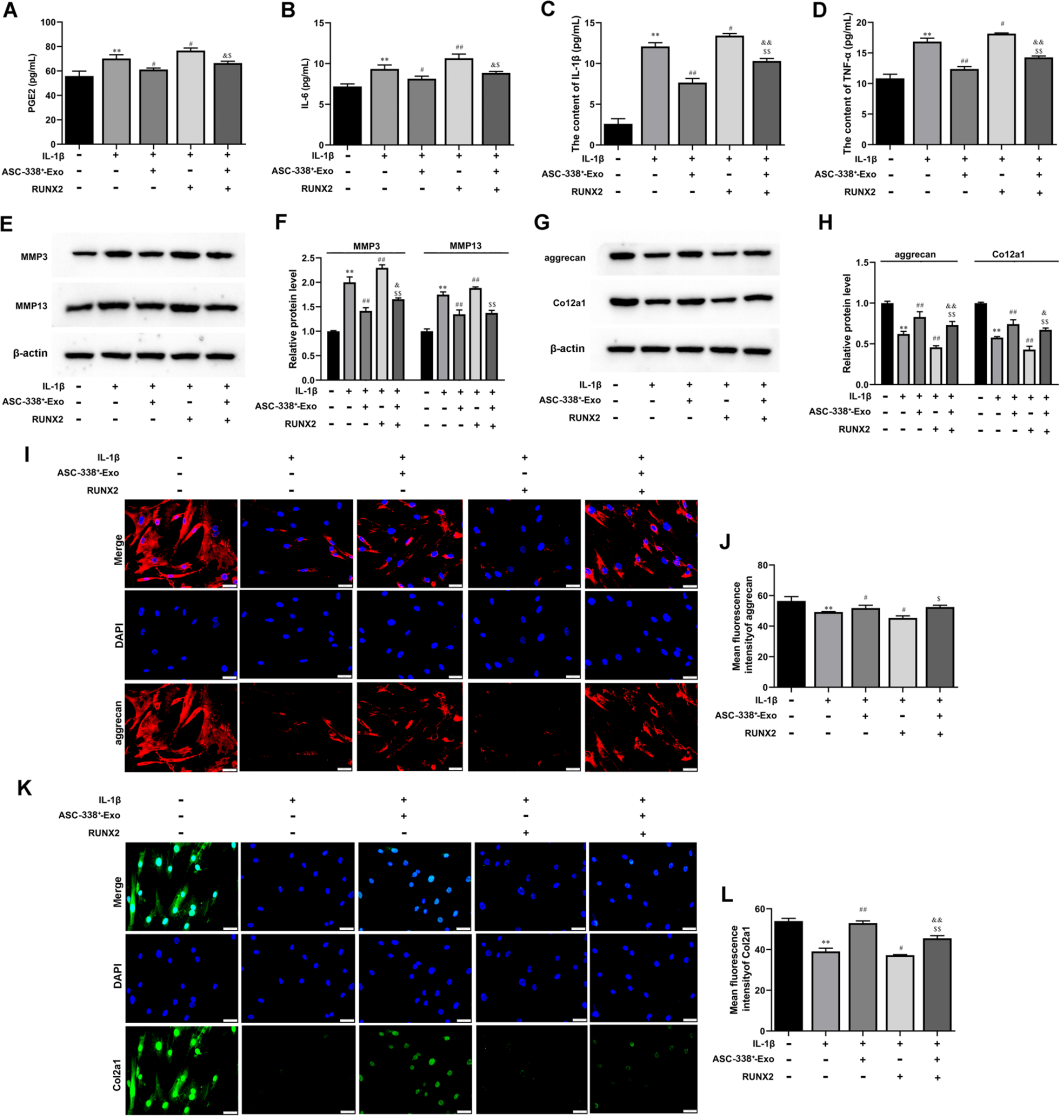
RUNX2 overexpression reversed ASC-338^+^-Exo-mediated attenuation of ATDC5 cell inflammation and degradation. **A–C** The expression levels of PGE2, IL-6, IL-1β, and TNF-αin ATDC5 cell supernatant were detected by ELISA. **D** Gene expression level of MMP3, MMP13, aggrecan, and Col2a1 in ATDC5 cells was determined by Western blot analysis. β-actin was used as the internal reference gene. **I–L** Immunofluorescence (IF) was used to test aggrecan and Col2a1 expression in ATDC5 cells. Nuclei were visualized using DAPI. Scale bar: 50 μm. ^**^P < 0.01 (VS. Blank), ^#^*P* < 0.05 (VS. IL-1β), ^##^*P* < 0.01 (VS. IL-1β), ^&^*P* < 0.05 (VS. IL-1β + ASC-388^+^-Exo), ^$^*P* < 0.05 (VS. IL-1β + RUNX2). ASC-338^+^-Exo, exosomes derived from miR-338-3p mimic-treated ASCs; RUNX2, pcDNA-RUNX2

ATDC5 cells were transfected with pcDNA-RUNX2, after which cells were pre-stimulated by IL-1β before being co-cultured with ASC-338^+^-Exos. RUNX2 overexpression significantly reversed ASC-338^+^-Exo-mediated decrease in the expression of PGE2, IL-6, IL-1β, and TNF-α (Fig. 7A–D). Furthermore, the decreased expression of MMP3 and MMP13 in ASC-338^+^-Exo-treated ATDC5 cells was enhanced by RUNX2 overexpression (Fig. 7E and F). Col2a1 and aggrecan were induced by ASC-338^+^-Exo, which was blocked by RUNX2 overexpression (Fig. 7G and L).

### RUNX2 overexpression impeded the regulatory effect of ASC-338^+^-Exo on ATDC5 cell proliferation and apoptosis

**Fig. 8:**
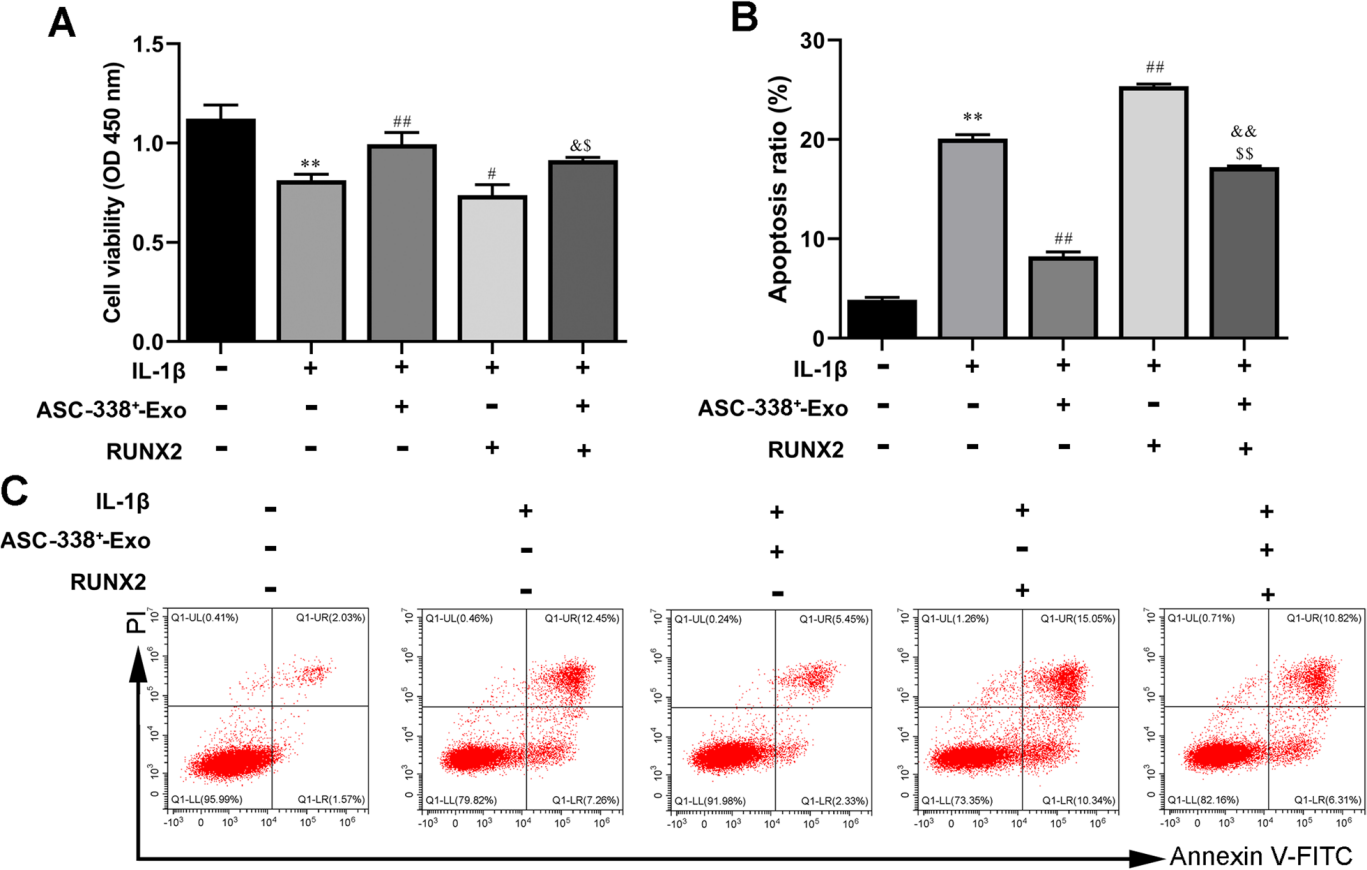
RUNX2 overexpression impeded the regulatory effect of ASC-338^+^-Exo on ATDC5 cell proliferation and apoptosis. **A** The cell proliferation of ATDC5 cells was tested by CCK-8 assay. **B** and **C** Flow cytometry analysis of apoptosis in ATDC5 cells. ^**^P < 0.01 (VS. Blank), ^#^*P* < 0.05 (VS. IL-1β), ^##^*P* < 0.01 (VS. IL-1β), ^&^*P* < 0.05 (VS. IL-1β + ASC-388^+^-Exo), ^$^*P* < 0.05 (VS. IL-1β + RUNX2). ASC-338^+^-Exo, exosomes derived from miR-338-3p mimic-treated ASCs; RUNX2, pcDNA-RUNX2

We also found that RUNX2 overexpression significantly hindered ASC-338+-Exo-mediated cell proliferation induction (Fig. 8A). Moreover, cell apoptosis of IL-1β-induced ATDC5 cells was inhibited by ASC-338+-Exo, which was further increased by RUNX2 overexpression (Fig. 8B–C). Together, these aforementioned results indicated that miR-338-3p derived from ASC-Exos involved in ASC-Exo-mediated chondrocyte proliferation induction via targeting RUNX2.

## Discussion

Chondrocyte proliferation promotion and inflammation inhibition have been the key strategies to prevent and control OA. In the present study, ASC-Exos promoted proliferation but inhibited inflammation and degradation of chondrocytes. Furthermore, the therapeutical effect of ASC-Exos on OA was related to novel exosomal miR-338-3p, which targeted RUNX2 mRNA 3’ UTR to inhibit RUNX2 expression in chondrocytes.

ASC-Exos have been reported to protect against spontaneous diabetes [32], tubulointerstitial fibrosis [33], histone-induced acute lung injury [34], atopic dermatitis [35], and more recently promote sciatic nerve [36], and bone regeneration [37]. In vivo, exosomes derived from infrapatellar fat pad MSCs maintained articular cartilage function and improved gait patterns of DMM-induced OA mice [38]. In addition, ASC-Exos suppressed inflammation‑inflicted oxidative stress of synovial fibroblasts and chondrocytes [39]. In this study, we successfully isolated ASC-Exos. And in ATDC5 cells stimulated with IL-1β, ASC-Exos overturned up-regulation of PGE2, IL-6, IL-1β, and TNF-α. Meanwhile, treatment of OA chondrocytes with ASC-Exos also enhanced the proliferation of the ATDC5 cells.

Several studies strongly supported that ASC-Exos is involved in the pathological regulation process by delivering functional miRNAs [40–42]. Exosomal miR-181-5p derived from ASCs effectively reduced CCl4-induced liver fibrosis [43]. And miR-199a-modified ASC-Exos improved hepatocellular carcinoma chemosensitivity via targeting mTOR signaling [44]. ASC-Exos reduced cell autophagy and promoted neurological recovery in stroke mice by extracellular vesicle transfer of miR-25 [45]. Interestingly, abundant miR-338-3p was first found in ASC-Exos by Marta García-Contreras et al. [26]. In the present experiments, we verified the presence of miR-338-3p in exosomes isolated from the supernatant of ASC culture. Meanwhile, there was exosome-mediated miR-338-3p transmission between ASCs and chondrocytes. Further ELISA analysis confirmed exosomes derived from miR-338-3p overexpression-modified ASCs could significantly inhibit the proliferation and inflammation of chondrocytes, compared with exosomes derived from untreated-ASCs. And exosomes derived from miR-338-3p silencing-modified ASCs showed the opposite results.

A previous clinical study revealed that the expression of miR-338-3p was increased in the serum of knee OA patients compared with normal controls and was positively correlated with visual analog scale (VAS) scores and joint space narrowing, indicating miR-338-3p may be as a promising biomarker for diagnosis of knee OA [46]. Furthermore, miR-338-3p in bone marrow mesenchymal stem cells (BMSCs) was reported to inhibit the differentiation of BMSCs into cartilage, characterized by decreased expression of SOX9, COL2, and Aggrecan [47]. However, in contradiction with these reports, our data demonstrated that miR-338-3p expression was decreased in IL-1β-stimulated chondrocytes. A study showed that in an acute liver injury model, miR-338-3p inhibited the expression of pro-inflammatory cytokines by preventing the nuclear factor kappa-B (NF-κB)/mitogen-activated protein kinase (MAPK) signaling pathway [48]. Similarly, we found miR-338-3p derived from ASC-Exos was beneficial to inhibit inflammation of chondrocytes. In short, the effects of different sources of miRNA-338-3p on osteoarthritis require further studies.

In addition, RUNX2 was identified as a target gene of miR-338-3p in the present study. It was discovered that in both murine models of OA and human patients, RUNX2 was highly expressed [49]. Furthermore, overexpression of RUNX2 in chondrocytes accelerates osteoarthritis progression in adult mice [50]. The deletion of RUNX2 in articular chondrocytes slows the progression of osteoarthritis induced by destabilization of the medial meniscus (DMM) in adult mice [51]. A recent study suggested that the accumulation of RUNX2 in articular chondrocytes and synoviocytes led to total joint degeneration [52]. RUNX2 has been reported to induce the expression of matrix-degrading enzymes such as MMP13 and a disintegrin-like and metalloproteinase with thrombospondin 4/5/7/12 (ADAMTS4/5/7/12), dramatically stimulating articular cartilage destruction, indicating the involvement of RUNX2 in cartilage degradation in OA [53–55]. RUNX2 directly regulated the expression of MMP13 and Adamts5 to digest collagen or aggrecan, respectively, thereby promoting articular cartilage degeneration during OA and resulting in impaired integrity of extracellular matrix (ECM) [56, 57]. Here, we observed that RUNX2 overexpression reversed miR-338-3p-Exos-mediated attenuation of chondrocyte inflammation and increase of chondrocyte proliferation induced by IL-1β.

This study has some limitations. This study is lack of the verification of animal experiment that should be addressed in future experiments. Furthermore, osteoclasts are also a major contributor to OA development [58, 59]. Thus, the effect of miR-338-3p-modified ASC-derived exosomes on osteoclast activity needs to be investigated in the future.

## Conclusions

Taken together, these results suggest that ASC-Exos derived exosomal miR-338-3p transplantation could inhibit chondrocyte inflammation and degradation, as well as promote chondrocyte proliferation through targeting RUNX2.

## Data Availability

The datasets used or analyzed during the current study are available from the corresponding author on reasonable request.

## Abbreviations

OA: Osteoarthritis
ASC: Adipose stem cells
MSCs: Mesenchymal stem cells
haMSCs: Human adipose-derived mesenchymal stem cells
BMSCs: Bone marrow mesenchymal stem cells
Exo: Exosome
IL: Interleukin
NF-κB: Nuclear factor kappa-B
MAPK: Mitogen-activated protein kinase
PGE2: Prostaglandin E2
MMP3: Matrix metalloproteinase3
TNF-α: Tumor necrosis factor-alpha
VAS: Visual analog scale

## References

[1] Sacitharan PK (2019). Ageing and Osteoarthritis. Subcell Biochem.

[2] Oliviero A, Della Porta G, Peretti GM, Maffulli N (2019). MicroRNA in osteoarthritis: physiopathology, diagnosis and therapeutic challenge. Br Med Bull.

[3] Gargano G, Oliva F, Oliviero A, Maffulli N (2022). Small interfering RNAs in the management of human rheumatoid arthritis. Br Med Bull.

[4] Attur M, Al-Mussawir HE, Patel J, Kitay A, Dave M, Palmer G, Pillinger MH, Abramson SB (1950). Prostaglandin E2 exerts catabolic effects in osteoarthritis cartilage: evidence for signaling via the EP4 receptor. J Immunol (Baltimore, MD: 1950).

[5] Kapoor M, Martel-Pelletier J, Lajeunesse D, Pelletier JP, Fahmi H (2011). Role of proinflammatory cytokines in the pathophysiology of osteoarthritis, nature reviews. Rheumatology.

[6] Wang T, He C (2018). Pro-inflammatory cytokines: The link between obesity and osteoarthritis. Cytokine Growth Factor Rev.

[7] Chien SY, Tsai CH (2020). Noggin inhibits IL-1β and BMP-2 expression, and attenuates cartilage degeneration and subchondral bone destruction in experimental osteoarthritis. J Cell.

[8] Wang J, Chen H, Cao P, Wu X, Zang F, Shi L, Liang L, Yuan W (2016). Inflammatory cytokines induce caveolin-1/β-catenin signalling in rat nucleus pulposus cell apoptosis through the p38 MAPK pathway. Cell Prolif.

[9] Ding DC, Shyu WC, Lin SZ (2011). Mesenchymal stem cells. Cell Transplant.

[10] McGonagle D, Baboolal TG, Jones E (2017). Native joint-resident mesenchymal stem cells for cartilage repair in osteoarthritis, nature reviews. Rheumatology.

[11] Uccelli A, Moretta L, Pistoia V (2008). Mesenchymal stem cells in health and disease. Nat Rev Immunol.

[12] Song Y, Du H, Dai C, Zhang L, Li S, Hunter DJ, Lu L, Bao C (2018). Human adipose-derived mesenchymal stem cells for osteoarthritis: a pilot study with long-term follow-up and repeated injections. Regen Med.

[13] Minteer D, Marra KG, Rubin JP (2013). Adipose-derived mesenchymal stem cells: biology and potential applications. Adv Biochem Eng Biotechnol.

[14] ter Huurne M, Schelbergen R, Blattes R, Blom A, de Munter W, Grevers LC, Jeanson J, Noël D, Casteilla L, Jorgensen C, van den Berg W, van Lent PL (2012). Antiinflammatory and chondroprotective effects of intraarticular injection of adipose-derived stem cells in experimental osteoarthritis. Arthritis Rheum.

[15] Pikuła M, Marek-Trzonkowska N, Wardowska A, Renkielska A, Trzonkowski P (2013). Adipose tissue-derived stem cells in clinical applications. Expert Opin Biol Ther.

[16] Zhang J, Li S, Li L, Li M, Guo C, Yao J, Mi S (2015). Exosome and exosomal microRNA: trafficking, sorting, and function. Genom Proteom Bioinform.

[17] Pegtel DM, Gould SJ (2019). Exosomes. Annu Rev Biochem.

[18] Kalluri R, LeBleu VS (2020). The biology, function, and biomedical applications of exosomes. Science.

[19] Yu B, Zhang X, Li X (2014). Exosomes derived from mesenchymal stem cells. Int J Mol Sci.

[20] Yaghoubi Y, Movassaghpour A, Zamani M, Talebi M, Mehdizadeh A, Yousefi M (2019). Human umbilical cord mesenchymal stem cells derived-exosomes in diseases treatment. Life Sci.

[21] Wang J, Guo X, Kang Z, Qi L, Yang Y, Wang J, Xu J, Gao S (2020). Roles of exosomes from mesenchymal stem cells in treating osteoarthritis. Cell Reprogram.

[22] Giordano L, Porta GD, Peretti GM, Maffulli N (2020). Therapeutic potential of microRNA in tendon injuries. Br Med Bull.

[23] Gargano G, Oliviero A, Oliva F, Maffulli N (2021). Small interfering RNAs in tendon homeostasis. Br Med Bull.

[24] Emanueli C, Shearn AI, Angelini GD, Sahoo S (2015). Exosomes and exosomal miRNAs in cardiovascular protection and repair. Vascul Pharmacol.

[25] Yang F, Ning Z, Ma L, Liu W, Shao C, Shu Y, Shen H (2017). Exosomal miRNAs and miRNA dysregulation in cancer-associated fibroblasts. Mol Cancer.

[26] García-Contreras M, Vera-Donoso CD, Hernández-Andreu JM, García-Verdugo JM, Oltra E (2014). Therapeutic potential of human adipose-derived stem cells (ADSCs) from cancer patients: a pilot study. PLoS ONE.

[27] Chen CC, Chen RF, Shao JS, Li YT, Wang YC, Brandacher G, Chuang JH, Kuo YR (2020). Adipose-derived stromal cells modulating composite allotransplant survival is correlated with B cell regulation in a rodent hind-limb allotransplantation model.

[28] Pak S, Hwang SW, Shim IK (2018). Endoscopic transplantation of mesenchymal stem cell sheets in experimental colitis in rats.

[29] Chen Y, Li K, Zhang X, Chen J, Li M, Liu L (2020). The novel long noncoding RNA lncRNA-Adi regulates adipogenesis.

[30] Oksvold MP, Neurauter A, Pedersen KW (2015). Magnetic bead-based isolation of exosomes. Methods Mol Biol (Clifton, N.J.).

[31] Hodax JK, Quintos JB, Gruppuso PA, Chen Q, Desai S, Jayasuriya CT (2019). Aggrecan is required for chondrocyte differentiation in ATDC5 chondroprogenitor cells. PLoS ONE.

[32] Jin J, Shi Y, Gong J, Zhao L, Li Y, He Q (2019). Exosome secreted from adipose-derived stem cells attenuates diabetic nephropathy by promoting autophagy flux and inhibiting apoptosis in podocyte.

[33] Chen L, Wang Y, Li S, Zuo B, Zhang X, Wang F, Sun D (2020). Exosomes derived from GDNF-modified human adipose mesenchymal stem cells ameliorate peritubular capillary loss in tubulointerstitial fibrosis by activating the SIRT1/eNOS signaling pathway. Theranostics.

[34] Mizuta Y, Akahoshi T (2020). Exosomes from adipose tissue-derived mesenchymal stem cells ameliorate histone-induced acute lung injury by activating the PI3K/Akt pathway in endothelial cells. Stem Cell Res Ther.

[35] Shin KO, Ha DH, Kim JO, Crumrine DA, Meyer JM, Wakefield JS, Lee Y, Kim B, Kim S, Kim HK, Lee J, Kwon HH, Park GH, Lee JH, Lim J, Park S, Elias PM, Park K, Yi YW, Cho BS (2020). Exosomes from human adipose tissue-derived mesenchymal stem cells promote epidermal barrier repair by inducing de novo synthesis of ceramides in atopic dermatitis. Cells.

[36] Chen J, Ren S, Duscher D, Kang Y, Liu Y, Wang C, Yuan M, Guo G, Xiong H, Zhan P, Wang Y, Machens HG, Chen Z (2019). Exosomes from human adipose-derived stem cells promote sciatic nerve regeneration via optimizing Schwann cell function.

[37] Xiong M, Zhang Q, Hu W, Zhao C, Lv W, Yi Y, Wu Y, Wu M (2020). Exosomes from adipose-derived stem cells: the emerging roles and applications in tissue regeneration of plastic and cosmetic surgery. Front Cell Develop Biol.

[38] Wu J, Kuang L, Chen C, Yang J, Zeng WN, Li T, Chen H, Huang S, Fu Z, Li J, Liu R, Ni Z, Chen L, Yang L (2019). miR-100-5p-abundant exosomes derived from infrapatellar fat pad MSCs protect articular cartilage and ameliorate gait abnormalities via inhibition of mTOR in osteoarthritis. Biomaterials.

[39] Zhao C, Chen JY, Peng WM, Yuan B, Bi Q, Xu YJ (2020). Exosomes from adipose-derived stem cells promote chondrogenesis and suppress inflammation by upregulating miR-145 and miR-221. Mol Med Rep.

[40] Bolandi Z, Mokhberian N, Eftekhary M, Sharifi K, Soudi S, Ghanbarian H, Hashemi SM (2020). Adipose derived mesenchymal stem cell exosomes loaded with miR-10a promote the differentiation of Th17 and Treg from naive CD4(+) T cell. Life Sci.

[41] Ching RC, Wiberg M, Kingham PJ (2018). Schwann cell-like differentiated adipose stem cells promote neurite outgrowth via secreted exosomes and RNA transfer. Stem Cell Res Ther.

[42] Storti G, Scioli MG, Kim BS, Orlandi A (2019). Adipose-derived stem cells in bone tissue engineering: useful tools with new applications.

[43] Qu Y, Zhang Q, Cai X, Li F, Ma Z, Xu M, Lu L (2017). Exosomes derived from miR-181-5p-modified adipose-derived mesenchymal stem cells prevent liver fibrosis via autophagy activation. J Cell Mol Med.

[44] Lou G, Chen L, Xia C, Wang W, Qi J, Li A, Zhao L, Chen Z, Zheng M, Liu Y (2020). MiR-199a-modified exosomes from adipose tissue-derived mesenchymal stem cells improve hepatocellular carcinoma chemosensitivity through mTOR pathway. J Exp Clin Cancer Res: CR.

[45] Kuang Y, Zheng X, Zhang L, Ai X, Venkataramani V, Kilic E, Hermann DM, Majid A, Bähr M, Doeppner TR (2020). Adipose-derived mesenchymal stem cells reduce autophagy in stroke mice by extracellular vesicle transfer of miR-25. J Extracellular Vesic.

[46] Dou X, Zhang Z, Wang S, Zhou X (2019). Combined use of serum miR-338-3p, cartilage oligomeric matrix protein and chondroitin sulfate-846 in the early diagnosis of knee osteoarthritis. Clin Lab.

[47] Zheng J, Lin Y, Tang F, Guo H, Yan L, Hu S, Wu H (2021). Promotive role of CircATRNL1 on chondrogenic differentiation of BMSCs mediated by miR-338-3p. Arch Med Res.

[48] Zhang C, Kang L, Zhu H, Li J, Fang R (2021). miRNA-338-3p/CAMK IIα signaling pathway prevents acetaminophen-induced acute liver inflammation in vivo. Ann Hepatol.

[49] Chen D, Kim DJ, Shen J, Zou Z, O'Keefe RJ (2020). Runx2 plays a central role in Osteoarthritis development. J Orthopaedic Transl.

[50] Catheline SE, Hoak D, Chang M, Ketz JP, Hilton MJ, Zuscik MJ, Jonason JH (2019). Chondrocyte-specific RUNX2 overexpression accelerates post-traumatic osteoarthritis progression in adult mice. J Bone Miner Res: Off J Am Soc Bone Miner Res.

[51] Liao L, Zhang S, Gu J, Takarada T, Yoneda Y, Huang J, Zhao L, Oh CD, Li J, Wang B, Wang M, Chen D (2017). Deletion of Runx2 in articular chondrocytes decelerates the progression of DMM-induced osteoarthritis in adult mice. Sci Rep.

[52] Huang J, Zhao L. The microRNAs miR-204 and miR-211 maintain joint homeostasis and protect against osteoarthritis progression, 2019;10, 2876. 10.1038/s41467-019-10753-5. 10.1038/s41467-019-10753-5 PMC6599052 31253842

[53] Wang X, Manner PA, Horner A, Shum L, Tuan RS, Nuckolls GH (2004). Regulation of MMP-13 expression by RUNX2 and FGF2 in osteoarthritic cartilage. Osteoarthritis Cartilage.

[54] Kamekura S, Kawasaki Y, Hoshi K, Shimoaka T, Chikuda H, Maruyama Z, Komori T, Sato S, Takeda S, Karsenty G, Nakamura K, Chung UI, Kawaguchi H (2006). Contribution of runt-related transcription factor 2 to the pathogenesis of osteoarthritis in mice after induction of knee joint instability. Arthritis Rheum.

[55] Ji Q, Xu X, Xu Y, Fan Z, Kang L, Li L, Liang Y, Guo J, Hong T, Li Z, Zhang Q, Ye Q, Wang Y (2016). miR-105/Runx2 axis mediates FGF2-induced ADAMTS expression in osteoarthritis cartilage. J Mol Med (Berl).

[56] Selvamurugan N, Jefcoat SC, Kwok S, Kowalewski R, Tamasi JA, Partridge NC (2006). Overexpression of Runx2 directed by the matrix metalloproteinase-13 promoter containing the AP-1 and Runx/RD/Cbfa sites alters bone remodeling in vivo. J Cell Biochem.

[57] Tetsunaga T, Nishida K, Furumatsu T, Naruse K, Hirohata S, Yoshida A, Saito T, Ozaki T (2011). Regulation of mechanical stress-induced MMP-13 and ADAMTS-5 expression by RUNX-2 transcriptional factor in SW1353 chondrocyte-like cells. Osteoarthritis Cartilage.

[58] Ibáñez L, Guillem-Llobat P, Marín M, Guillén MI. Connection between mesenchymal stem cells therapy and osteoclasts in osteoarthritis, 2022;23. 10.3390/ijms23094693. 10.3390/ijms23094693 PMC9102843 35563083

[59] PloS ONE. 10.1371/journal.pone.0261127.

